# Mapping nationally adopted targets for antimicrobial consumption and stewardship indicators in the human health domain, 25 European Union/European Economic Area countries, 2025

**DOI:** 10.2807/1560-7917.ES.2026.31.34.2500976

**Published:** 2026-08-27

**Authors:** Liselotte Diaz Högberg, Vera Vlahović-Palčevski, Vivian H Leung, ESAC-Net study group, ESAC-Net study group, Julia Weber, Lucy Catteau, Arjana Tambic Andrasevic, Lenka Vostalová, Majda Attauabi, Janne Sepp, Emmi Sarvikivi, Ghaya Ben Hmidene, Imke Wieters, Lida Politi, Ria Benko, Anna Margrét Halldórsdóttir, Marie Philbin, Filomena Fortinguerra, Rolanda Valintėlienė, Stéphanie Saleh, Peter Zarb, Jan Baltink, Hege Salvesen, Aneta Mroczkowska, Joana Fernandes, Gabriel-Adrian Popescu, Tomas Tesar, Maja Subelj, Ragda Obeid

**Affiliations:** 1 European Centre for Disease Prevention and Control (ECDC), Stockholm, Sweden; 2 Clinical Hospital Center Rijeka and University of Rijeka Medical Faculty, Rijeka, Croatia; 3 The members of the ESAC-Net study group are listed under Collaborators

**Keywords:** antimicrobial consumption, antimicrobial stewardship, targets, Europe

## Abstract

**BACKGROUND:**

Establishing quantifiable targets for antimicrobial consumption (AMC) and stewardship activities can provide a structured framework for monitoring and evaluating progress in efforts to curb antimicrobial resistance (AMR).

**AIM:**

We aimed to map the presence and characteristics of national AMC-related targets in the human health domain across European Union (EU)/European Economic Area (EEA) countries, thereby creating an inventory to inform current and future target-setting initiatives.

**METHODS:**

We performed a survey within the European Surveillance of Antimicrobial Consumption Network (ESAC-Net) to identify national AMC-related targets in force for 2025 or later.

**RESULTS:**

Based on responses received from 25 EU/EEA countries, we identified a total of 67 nationally adopted AMC-related targets, active for 2025 or beyond, from 16 countries. Targets for population-level AMC indicators were the most frequently reported (73%), followed by patient-level AMC indicators (18%) and structural stewardship indicators (9%). The influential role of the 2023 EU Council Recommendation on AMR and its recommendations for national targets was particularly evident in our findings.

**CONCLUSION:**

This inventory provides examples of national targets and related implementation and monitoring frameworks used across a range of settings and resource levels. Our findings highlight the importance of robust and sustainable surveillance infrastructure and reporting systems to support the development, implementation, monitoring and evaluation of AMC-related national targets. Such systems are also needed to enable targets that capture prescribing practices and antimicrobial stewardship activities, beyond overall consumption volume metrics alone.

**Key public health message**

**What did you want to address in this study and why?**

Targets for antimicrobial consumption (AMC) and antimicrobial stewardship (AMS) can support national efforts to control antimicrobial resistance (AMR). We aimed to identify which EU/EEA countries had national targets for AMC and AMS in place in the human health domain. By providing an overview of existing targets, this work is intended to support countries in developing or refining their AMC-related target-setting efforts.

**What have we learnt from this study?**

Based on responses from 25 of the 30 EU/EEA countries, most (16/25 countries) had established national AMC targets for 2025 or beyond, with the majority focused on reducing overall consumption volumes. The 2023 EU AMR Council Recommendation, which encourages countries to take appropriate national measures to ensure that specific EU-level AMC targets can be met by 2030, appeared to be a key driver for the most recent target developments.

**What are the implications of your findings for public health?**

Continued investment in AMC surveillance and AMS programme monitoring remains essential to enable more context-specific targets supporting improved prescribing practices and stewardship activities.

## Introduction

Antimicrobial resistance (AMR) is considered one of the main threats to public health in the European Union (EU)/ European Economic Area (EEA) [1] and globally [2]. Setting concrete and measurable targets for antimicrobial consumption (AMC) and antimicrobial stewardship (AMS) indicators can improve accountability and support the implementation, monitoring and evaluation of national efforts to control AMR [3,4].

The 2023 European Council Recommendation on stepping up EU Actions to combat AMR in a One Health approach encourages EU countries to take appropriate national measures to ensure that specific EU-level and national targets for AMC in humans can be met by 2030 [1]. Using 2019 as a baseline year, this calls for a 20% EU-level reduction in the consumption of antibacterials for systemic use as defined by the Anatomical Therapeutic Chemical (ATC) classification system group J01. Corresponding recommendations for country-specific reductions range from 3% to 27% based on the respective levels of national antimicrobial consumption in 2019, and reflect the effort required by each country to reach the EU goal. In addition, all countries are expected to achieve the EU target of ensuring that at least 65% of total antimicrobial consumption consists of antimicrobials belonging to the Access group of the World Health Organization (WHO) Access, Watch and Reserve (AWaRe) antimicrobial classification system [5] by 2030. The EU target for WHO AWaRe Access group consumption was set higher than the global target of at least 60% as defined by the WHO at the time [6], but is now lower than the 70% threshold suggested at the United Nations General Assembly (UNGA) High-Level Meeting on Antimicrobial Resistance in 2024 [2]. Both the EU Council Recommendation and the UNGA Declaration encourage countries to develop additional national indicators and targets appropriate to local contexts and needs.

Studies from 2017 concluded that although national targets for AMC and AMS in the human health domain were not commonly used in the EU/EEA at that time, several countries were in the process of establishing such targets for the coming years [7,8]. To the best of our knowledge, no recent overview of the current uptake of national targets in EU/EEA countries is available. We therefore conducted a survey within the European Surveillance of Antimicrobial Consumption Network (ESAC-Net) to map the current presence of national targets for AMC and AMS in the human health domain across EU/EEA countries in order to provide an inventory supporting ongoing and future national target-setting efforts.

## Methods

Authors from the European Centre for Disease Prevention and Control (ECDC) drafted a short survey that was reviewed and finalised in collaboration with the ESAC-Net Disease Network Coordination Committee. The survey is available as Supplementary material.

In March 2025, ECDC requested the National Focal Points for AMC from each EU/EEA country to nominate one ESAC-Net representative to provide information on the current presence of national targets related to AMC and AMS in the human health domain. Nominations, consisting of persons serving as National Focal Points or Operational Contact Points for AMC, were received from 28 EU/EEA countries (Austria, Belgium, Bulgaria, Croatia, Czechia, Cyprus, Denmark, Estonia, Finland, France, Germany, Greece, Hungary, Iceland, Ireland, Italy, Lithuania, Luxembourg, Malta, the Netherlands, Norway, Poland, Portugal, Romania, Slovakia, Slovenia, Spain and Sweden). In May 2025, ECDC e-mailed a questionnaire to the nominated persons, followed by a maximum of three email reminders per country if required. By the end of August 2025, survey responses were received from 25 countries. Between August and the finalisation of the manuscript in November 2025, Estonia, Finland, Slovakia and Sweden updated their information to reflect changes in target frameworks (national AMR action plan, AMR strategy or similar) that had taken place since the time of the survey.

The term ‘AMC-related’ is hereafter used for indicators related to AMC, antimicrobial use and AMS in the human health domain. The authors reviewed the survey responses, defining an AMC-related target as either (i) a predefined and quantifiable change in an AMC-related indicator within a specific time frame, (ii) a predefined maximum or minimum threshold value to be met within or without a specified time frame or (iii) a rolling target relative to performance in the previous year or at EU/EEA area level to which performance was monitored continuously. The target should be included in a published or forthcoming AMR national action plan (NAP), strategy or similar official framework, in force for 2025 or later.

The AMC-related targets were categorised into three groups based on their related indicators: (i) population-level targets for absolute or relative AMC without further specification; (ii) patient-level targets for absolute or relative AMC defined by patient demographics (age, sex), clinical or prescription information (indication for use, length of treatment), and (iii) structural AMS targets aiming to strengthen the infrastructures, systems, and practices that support prescribers and healthcare institutions in achieving AMC-related goals.

## Results

### Overall findings

Of the 25 countries responding to our survey, sixteen countries (Belgium, Finland, France, Germany, Greece, Iceland, Ireland, Italy, Lithuania, Luxembourg, Malta, the Netherlands, Norway, Portugal, Slovakia and Sweden) reported a total of 67 AMC-related targets that met our inclusion criteria. Of the remaining nine responding countries, four reported plans to include AMC-related targets in their national frameworks in the coming years (Croatia, Czechia, Estonia and Poland), two reported frameworks with formalised monitoring of AMC-related indicators and established progress evaluation components but without any quantifiable targets (Austria and Denmark) and one reported continued monitoring of recently expired targets (Slovenia). An overview of the targets reported in our study is presented in Table 1, with the target indicators presented in more detail in Tables 2,3,4. A summary of survey responses by country and additional contextual information provided by some survey respondents is available as Supplementary material.

**Table 1:** Overview of survey responses: number of reported national antimicrobial consumption-related targets in the human health domain, active for 2025 or later, by country and target characteristics^a^, 25 European Union/European Economic Area countries

Country	Remarks									
	Target category^a^ and healthcare sector attribution								Target endpoint	Framework for establishment
	Population-level AMC targets^a^				Patient-level AMC targets^a^		Structural AMS targets^a^			
	Total^b^	Community	Hospital	LTCF	Community	Hospital	Total^b^	Hospital		
Belgium	–	2	–	–	2	–	–	–	Continuous monitoring thresholds	Guidance from the National Institute for Health and Disability Insurance [18]
	–	–	–	–	–	–	–	1		Royal decree [19]
Finland	2	–	–	–	–	–	–	–	2030	National Action Plan for Antimicrobial Resistance, Implementation programme for the years 2025–2028 and plan for the year 2025 [20]
France	1	2	2	1	1	1	–	–	2027 (extended from 2025)	National Strategy on Prevention of Infections and Antibiotic Resistance 2022–2025 [21]
Germany	2	–	–	–	–	–	–	–	2030	Action plan for German Antimicrobial Resistance Strategy (DART 2030), 2024–2026 [22]
Greece^c^	2	–	–	–	–	–	–	–	2030	Forthcoming AMR NAP
Iceland	2	2	2	–	–	–	3	–	2029	National Action Plan on Antimicrobial Resistance 2025–2029 [23]
Ireland	–	3	1	–	–	1	–	–	2025	Irish Health Service Antimicrobial Resistance and Infection Control Action Plan 2022–2025 [10]
Italy	–	2	3	–	3	–	–	–	2025	National Plan to Combat Antibiotic Resistance (PNCAR) 2022–2025 [24]
	1	–	1	–	–	–	–	–	2030	Report on Antibiotics Use from the Italian Medicines Agency [25]
Lithuania	–	–	–	–	–	–	–	1	2028	Governmental implementation plan [26]
Luxembourg^c^	2	–	–	–	–	–	–	–	2030	Forthcoming National Antibiotics Plan.
Malta	2	–	1	–	–	–	–	–	2030	A Strategy and Action Plan for the Prevention and Containment of Antimicrobial Resistance in Malta (2020 – 2028) [27]
The Netherlands	2	–	–	–	–	–	–	–	2030	The Netherlands’ Action Plan for Reducing Antimicrobial Resistance 2024–2030 [28]
Norway	1	–	–	–	–	–	–	–	2033	Norwegian National One Health Strategy Against Antimicrobial Resistance 2024–2033 [29]
	–	1	–	–	–	–	–	–	Continuous monitoring threshold	National Strategy against Antibiotic Resistance 2015–2020 [30]
Portugal	–	2	2	–	–	–	–	–	Continuous monitoring thresholds	Governmental decrees for the Programme for the Prevention and Control of Infections and Resistance to Antimicrobials PPCIRA [31,32]
Slovakia^c^	2	–	–	–	–	–	–	–	2030	Forthcoming AMR NAP
Sweden	1	–	–	–	–	–	–	–	2030	Swedish Strategy to Combat Antibiotic Resistance 2026–2035 [33]
	1	1	–	–	–	–	–	–	2035	
	–	–	–	–	2	2	–	1	Continuous monitoring thresholds	Guidance by the Swedish Strategic Programme against antibiotic resistance (STRAMA) [34,35]
**Total number of targets**	**21**	**15**	**12**	**1**	**8**	**4**	**3**	**3**		
Countries reporting no presence of AMC-related targets as defined by the study’s inclusion criteria										
Austria	Formalised national monitoring of antimicrobial stewardship indicators through the Quality standard for anti-infectives [36], but without defined target values.									
Croatia	Reduction in total AMC is included as an indicator in the current AMR national action plan but without a defined target value. Specific targets will however be considered in the next update of the National Programme for the Control of Antibiotic-Resistant Bacteria.									
Czechia	No AMC-related targets were reported from Czechia. However, the Strategy of the National Antibiotic Programme of the Czech Republic for the period 2025–2031, currently under development, is expected to define goals for reducing antibiotic consumption in line with international recommendations.									
Denmark	Formalised national monitoring of AMC-related indicators in the AMR NAP [37], but without defined target values.									
Estonia	Currently no national AMC-related targets, but considerations for their inclusion in the upcoming AMR NAP.									
Hungary	No national AMC-related targets were reported. However, the national One Health strategy to combat Antimicrobial Resistance, currently under approval, is expected to include indicators promoting the prudent use of antimicrobials.									
Poland	Currently no national AMC-related targets, but considerations for inclusion in the upcoming AMR NAP.									
Romania	No national AMC-related targets were reported.									
Slovenia	Continued monitoring of targets included in the recently expired National One Health Strategy for the Management of Microbial Resistance 2019–2024 [38].									

AMC: antimicrobial consumption; AMR: antimicrobial resistance; LTCF: long-term care facilities; NAP: national action plan; –: None.

^a^ Population-level AMC targets: absolute or relative AMC without further specification; Patient-level AMC targets: absolute or relative AMC defined by patient demographics (age, sex), clinical or prescription information (indication for use, length of treatment); Structural AMS targets: indicators related to the infrastructures, systems and practices that support prescribers and healthcare institutions in achieving AMC-related goals.

^b^ All healthcare sectors combined.

^c^ Targets not yet officially published but are expected in the forthcoming AMR NAP.

More information on target frameworks, monitoring and evaluation components is available as Supplementary material.

**Table 2:** National population-level antimicrobial consumption targets, by indicator type and healthcare sector, European Union/European Economic Area countries, 2025

Consumption targets		Source
Total care (all healthcare sectors combined)		
Targets for absolute consumption of all antibacterials for systemic use (ATC J01 or similar adaptations)	Decrease by ≥ 3% DDD/1,000 inhabitants/day, ATC J01, 2019–2030, according to the country-specific recommendations in the EU Council Recommendation [1].	The Netherlands [28] and Sweden [33]
	Decrease by ≥ 9% DDD/1,000 inhabitants/day, ATC J01, 2019–2030, according to the country-specific recommendations in the EU Council Recommendation [1].	Germany [22], Finland [20] and Slovakia^a^
	Decrease by ≥18% DDD/1,000 inhabitants/day, ATC J01, 2019–2030, according to the country-specific recommendations in the EU Council Recommendation [1].	Luxembourg^a^ and Malta [27]
	Decrease by ≥ 27% DDD/1,000 inhabitants/day, ATC J01, 2019–2030, according to the country-specific recommendation in the EU Council Recommendation [1].	Greece^a^
	Decrease by ≥ 10% DDD/1,000 inhabitants/day, ATC J01 excluding J01XX05, 2019–2033.	Norway [29]
	End-point value of < 20 DDD/1,000 inhabitants/day, ATC J01, by 2027.	France [21]
	End-point value of ≤ 15 DDD/1,000 inhabitants/day, ATC J01, by 2029.	Iceland [23]
Targets for relative consumption of selected antibacterial subgroups	End-point value of ≥ 65% of WHO AWaRe Access group consumption, DDD/1,000 inhabitants/day, by 2030, as recommended by the EU Council Recommendation [1].	Finland [20], Germany [22], Greece^a^, Italy [25], Luxembourg^a^, Malta [27], the Netherlands [28] and Slovakia^a^
	End-point value of ≥ 75% of WHO AWaRe Access group consumption, DDD/1,000 inhabitants/day, by 2035.	Sweden [33]
	End-point value of > 80% of WHO AWaRe Access group consumption, DDD/1,000 inhabitants/day, by 2029.	Iceland [23]
Community (primary care) sector		
Targets for absolute consumption of all antibacterials for systemic use (ATC J01 or similar adaptations)	Decrease by ≥ 10% in the DDD/1,000 inhabitants/day, ATC J01, 2022–2025.	Italy [24]
	End-point value of ≤ 14 DDD/1,000 inhabitants/day, ATC J01, by 2029.	Iceland [23]
	End-point value of < 20.5 DDD/1,000 inhabitants/day, ATC J01, by 2025.	Ireland [10]
	End-point value of < 101 prescriptions /100 medical card patients /year, ATC J01, by 2025.	Ireland [10]
	End-point value of ≤ 250 prescriptions/1,000 inhabitants/year, ATC J01 excluding J01XX05, by 2035.	Sweden [33]
	End-point value of < 650 prescriptions/1,000 inhabitants /year, ATC J01, by 2027.	France [21]
	Threshold of < DDD/1,000 inhabitants/day as compared with the previous year, ATC J01, continuous monitoring.	Portugal [31,32]
	Threshold of ≤ 250 prescriptions/1,000 inhabitants/year, ATC J01 excluding J01XX05, continuous monitoring.	Norway [30]
Targets for absolute consumption of selected antibacterial subgroups	Decrease by > 20% in the DDD/1,000 inhabitants/day, SPILF critical antibiotics for systemic use [9], 2019–2027.	France [21]
	End-point value of < 2.5 DDD/1,000 inhabitants/day, tetracyclines (ATC J01A), by 2029.	Iceland [23]
Targets for relative consumption of selected antibacterial subgroups	Decrease by ≥ 20% in the ratio of the ECDC, EFSA and EMA secondary AMC indicator for the community sector [11], DDD/1,000 inhabitants/day, 2022–2025.	Italy [24]
	End-point value of < 26% of WHO AWaRe Watch and Reserve group consumption (based on a modified AWaRe list), by 2025.	Ireland [10]
	Threshold of < ratio of the ECDC, EFSA and EMA secondary AMC indicator for the community sector [11] as compared with the previous year, DDD/1,000 inhabitants/day, continuous monitoring.	Portugal [31,32]
	Threshold of ≥ 80% amoxicillin (ATC J01CA04) out of the total DDDs for amoxicillin (J01CA04) and amoxicillin with beta-lactamase inhibitor (J01CR02) combined, continuous monitoring.	Belgium [18]
	Threshold of ≤ 20% second-line antibiotics (amoxicillin combined with clavulanic acid ATC J01CR02, cephalosporins J01D, quinolones J01M and macrolides J01FA) out of the total DDD for antibacterials for systemic use (J01), continuous monitoring.	Belgium [18]
Hospital sector		
Targets for absolute consumption of all antibacterials for systemic use (ATC J01 or similar adaptations)	Decrease by ≥ 10% in the DDD dispensed in hospitals to hospitalised patients/1,000 hospital days, ATC J01 + J04AB02 + P01AB + A07AA12, 2019–2027.	France [21]
	Decrease by ≥ 5% in the DDD/100 hospital days, ATC J01, 2022–2025.	Italy [24]
	End-point value of ≤ 1.0 DDD/1,000 inhabitants/day, ATC J01, by 2029.	Iceland [23]
	End-point value of < 72.1 DDD/100 bed-days used/day, ATC J01, by 2025.	Ireland [10]
	Threshold of < 5000 DDD/1,000 patient discharges or a reduction of at least 20% compared with the previous year, ATC J01, continuous monitoring.	Portugal [31,32]
Targets for absolute consumption of selected antibacterial subgroups	Decrease by ≥ 10% in the DDD/100 hospitalisation days, fluoroquinolones (ATC J01MA), 2022–2025.	Italy [24]
	Decrease by ≥ 10% in the DDD/100 hospitalisation days, carbapenems (ATC J01DH), 2022-2025.	Italy [24]
	End-point value of ≤ 0.15 DDD/1,000 inhabitants/day, third-generation cephalosporins (ATC J01DD), by 2029.	Iceland [23]
	Threshold of < 200 DDD/1,000 patient discharges or a decrease by > 20% compared with the previous year, carbapenems (ATC J01DH), continuous monitoring.	Portugal [31,32]
Targets for relative consumption of selected antibacterial subgroups	Decrease by ≥ 10% in the proportion of broad-spectrum antibiotics (third- and fourth generation cephalosporins, piperacillin-tazobactam, aztreonam, carbapenems, fluoroquinolones, glycopeptides, linezolid, tedizolid, daptomycin and colistin) out of total hospital consumption of antibacterials for systemic use (ATC J01), 2019 -2027.	France [21]
	Threshold of < national value compared with the population-weighted EU/EEA mean of the ECDC, EFSA, EMA secondary AMC indicator for the hospital sector [11] as reported by ECDC ESAC-Net, continuous monitoring.	Malta [27]
	Threshold of < national value compared with the population-weighted EU/EEA mean of the ECDC, EFSA, EMA secondary AMC indicator for the hospital sector [11] as reported by ECDC ESAC-Net, continuous monitoring.	Italy [25]
Long-term care facilities		
Targets for absolute consumption or selected antibacterial subgroups	Decrease by > 20% in the DDD/1,000 LTCF residents/year or per 1,000 days of hospitalisation/year, SPILF critical antibiotics for systemic use [9] dispensed by community pharmacies and in-house pharmacies for residents in LTCF, 2019–2027.	France [21]

ATC: Anatomical Therapeutic Chemical classification system; AWaRe: Access, Watch Reserve classification system [5]; DDD: defined daily dose; ECDC: European Centre for Disease Prevention and Control; EEA: European Economic Area; EFSA: European Food Safety Authority; EMA: European Medicines Agency; ESAC-Net: European Surveillance of Antimicrobial Consumption network; EU: European Union; LTCF: long-term care facility; SPILF: Société de Pathologie Infectieuse de Langue Française; WHO: World Health Organization.

^a^ Publication of target framework forthcoming. More information on target frameworks, monitoring and evaluation components is available as Supplementary material.

**Table 3:** National patient-level antimicrobial consumption targets, by healthcare sector and indicator type, European Union/European Economic Area countries, 2025

Consumption targets		Source
Community (primary care) sector		
Targets for absolute consumption of all antibacterials for systemic use (ATC J01 or similar adaptations)	Decrease by ≥ 10% in the number of packages dispensed for children aged 0–13 years /1,000 inhabitants/year, ATC J01, 2022-2025.	Italy [24]
	End-point value of < 20 prescriptions/100 patients aged 16–65 years with no long-term illness and attending a physician, ATC J01, by 2027.	France [21]
	Threshold of ≤ 45% prescriptions out of all reimbursed pharmaceutical prescriptions for paediatric patients aged < 15 years, ATC J01, continuous monitoring.	Belgium [18]
	Threshold of ≤ 23% prescriptions out of all reimbursed pharmaceutical prescriptions for adult patients aged ≥ 15 years, ATC J01, continuous monitoring.	Belgium [18]
Targets for relative consumption of selected antibacterial subgroups or clinical conditions	Decrease by ≥ 20% in the ratio of the ECDC, EFSA and EMA secondary AMC indicator for the community sector [11], number of packages/1,000 inhabitants/year, children aged 0–13 years, 2022 - 2025.	Italy [24]
	Increase by ≥ 30% in the ratio of amoxicillin (ATC J01CA04) to amoxicillin and clavulanic acid (J01CR02), number of packages/1,000 inhabitants/year, children aged 0–13 years, 2022 - 2025.	Italy [24]
	Threshold of ≥ 80% phenoxymethylpenicillin (ATC J01CE02) prescriptions out of the antibiotics prescribed for respiratory tract infections in (including penicillin J01CE02, amoxicillin J01CA04, amoxicillin with enzyme inhibitor J01CR02, cephalosporins J01DB-DE and macrolides J01FA), prescriptions /1,000 inhabitants/year, children aged 0–6 years, continuous monitoring.	Sweden [34]
	Threshold of ≤ 10% ciprofloxacin (ATC J01MA02) and norfloxacin (J01MA06) prescriptions out of the antibiotics commonly prescribed to treat urinary tract infections (including pivmecillinam J01CA08, trimethoprim J01EA01, ciprofloxacin J01MA02, norfloxacin J01MA06 and nitrofurantoin J01XE01), prescriptions /1,000 inhabitants/year in women aged 18–79 years, continuous monitoring.	Sweden [34]
Hospital sector		
Targets for relative consumption of selected antibacterial subgroups or clinical conditions	End-point value of ≥ 80% of antibiotic prescriptions for lower respiratory tract infections having a treatment duration shorter than 7 days, number of compliant health records/total health records, by 2027.	France [21]
	End-point value of ≤ 20% of surgical antibiotic prophylaxis prescriptions extended beyond 24 hours, annual national point prevalence survey, by 2025.	Ireland [10]
	Threshold of < 10% of quinolones (ATC J01MA) in female inpatients out of the antibiotics used to treat afebrile urinary tract infections in inpatients (fluoroquinolones J01M), pivmecillinam J01CA08, nitrofurantoin J01XE01 and trimethoprim J01EA01, continuous monitoring threshold.	Sweden [35]
	Threshold of < 20% of quinolones (ATC J01MA) in male inpatients out of the antibiotics used to treat afebrile urinary tract infections in inpatients (fluoroquinolones J01MA, pivmecillinam J01CA08, nitrofurantoin J01XE01 and trimethoprim J01EA01, continuous monitoring.	Sweden [35]

ATC: Anatomical Therapeutic Chemical classification system; ECDC: European Centre for Disease Prevention and Control; EFSA: European Food Safety Authority; EMA: European Medicines Agency.

More information on target frameworks, monitoring and evaluation components is available as Supplementary material.

**Table 4:** National structural antimicrobial stewardship targets, by healthcare sector and indicator type, European Union/European Economic Area countries, 2025

Consumption targets		Source
Total care (all healthcare sectors combined)		
Targets for stewardship programmes	End-point value of > 95% of health institutions having an active antibiotic stewardship program, by 2029.	Iceland [23]
Targets for surveillance system capacity	End-point value of > 90% of all antibiotic prescriptions in humans being recorded in a central database, by 2029.	Iceland [23]
Targets for treatment guideline availability	End-point value of > 95% of a defined target group (larger hospitals, nursing homes, primary care centres, and specialists outside hospitals who prescribe antibiotics the most) having specific guidelines for targeted and prudent use of antibiotics, by 2029.	Iceland [23]
Hospital sector		
Targets for stewardship programmes	Threshold of 100% of the hospitals with ≥ 150 beds having an antimicrobial stewardship programme, continuous monitoring.	Belgium [19]
	End-point value of ≥ 85% of hospitals having an antimicrobial stewardship programme, by 2028.	Lithuania [26]
	Threshold of ≥ 0.1 full-time equivalent personnel dedicated to antibiotic stewardship work/100 beds, continuous monitoring.	Sweden [35]

More information on target frameworks, monitoring and evaluation components is available as Supplementary material.

Targets originated from AMR NAPs, related implementation plans and strategies, official decrees or the work of other national AMR initiatives such as the Belgian National Institute for Health and Disability Insurance, the Portuguese Infection Prevention and Control and Antimicrobial Resistance Programme and the Swedish Strategic Programme Against Antibiotic Resistance. The majority of targets were supported by already published frameworks, while three countries (Greece, Luxembourg and Slovakia) reported a total of six targets expected in forthcoming publications (Table 1).

Population-level AMC targets were the most frequently reported (49/67, 73%), followed by patient-level AMC targets (12/67, 18%) and structural AMS targets (6/67, 9%). Targets for specific healthcare sectors (community, hospital and long-term care facilities) were more common than targets applying to the total care sector (all healthcare sectors combined). The distribution of target categories varied between sectors. Targets for the total care sector only covered population-level AMC and structural AMS indicators, targets for the community sector only included population-level and patient-level AMC indicators, while targets reported for the hospital sector included all three categories. One population-level AMC target was reported for the long-term care facility sector specifically (Table 1).

Most target outcomes were time-bound and defined as either a proportional change within a specified time period or as an end-point value to be achieved by a designated year. Target end-years ranged between 2025 and 2035. The targets that did not have a specified end-year were instead monitored continuously against a fixed or rolling threshold value. These thresholds were either expressed as static maximum or minimum values or quantified the target level as relative to national performance in the previous year or to the EU/EEA population-weighted mean value reported by ECDC ESAC-Net (Tables 1–4).

### Population-level antimicrobial consumption targets

Fifteen countries reported a total of 49 national population-level AMC targets, with indicators including both absolute and relative AMC. Absolute AMC focused on overall consumption of antibacterials for systemic use (ATC J01 or similar national adaptations), ‘critical antimicrobials’ as defined nationally [9] or specific ATC subgroups including carbapenems (J01DH), fluoroquinolones (J01MA), third-generation cephalosporins (J01DD) and tetracyclines (J01A). Target indicators for relative AMC included proportions of specific WHO AWaRe categories [5] or similar national adaptations [10] and proportions or ratios of narrow- to broad-spectrum agents based on the ECDC, European Food Safety Authority (EFSA) and European Medicines Agency (EMA) secondary AMC indicators for the community and hospital sectors [11] or a variety of national definitions. Various indicator metrics were applied, with AMC quantified through defined daily doses (DDD) or the number of prescriptions and packages, and denominators based on number of inhabitants or healthcare utilisation such as patient visits, medical records, hospital bed-days, and hospital discharges (Table 2).

Sixteen of the national population-level targets reported by EU countries were direct adoptions of the targets suggested by the EU Council Recommendation [1]. This included the country-specific reduction targets for total consumption of antibacterials for systemic use (ATC J01) reported by Finland, Germany, Greece, Luxembourg, Malta, the Netherlands, Slovakia and Sweden, and the threshold of at least 65% of WHO AWaRe Access group antimicrobials reported by Finland, Germany, Greece, Italy, Luxembourg, Malta, the Netherlands and Slovakia. Sweden reported an end-point target of 75% WHO AWaRe Access group antimicrobials by 2035, which is higher than that prescribed by the EU Council Recommendation but with a 5-year longer period until the target endpoint (Table 2).

### Patient-level antimicrobial consumption targets

Four countries reported a total of 12 national patient-level AMC targets, covering specific populations defined by age, sex, clinical presentation or treatment indications such as respiratory tract infections, urinary tract infections or surgical prophylaxis. Antimicrobial consumption was quantified as the number of antimicrobial prescriptions or packages, and denominators were based on either the number of inhabitants or healthcare utilisation data such as patient visits, prescriptions or medical records (Table 3).

For the community sector, targets for absolute AMC addressed overall consumption of antibacterials for systemic use (ATC J01) for adults and children separately. Targets for relative AMC mainly focused on children and included proportions or ratios of narrow- to broad-spectrum agents based on the ECDC, EFSA and EMA secondary AMC indicator for the community sector [11] or groups of antimicrobials commonly used to treat respiratory tract infections. One target for relative AMC in the community sector covered adults, setting a threshold for the proportion of macrolide consumption among the antibiotics commonly prescribed for urinary tract infections in adult females (Table 3).

For the hospital sector, all targets were based on relative AMC. These included threshold values for the proportions of antimicrobial prescriptions for lower respiratory tract infections and for surgical prophylaxis within a specific treatment duration, and the proportions of macrolide AMC in female and male patients with afebrile urinary tract infections (Table 3).

### Structural antimicrobial stewardship targets

Four countries reported a total of six structural AMS targets at national level, including requirements for AMS programmes, surveillance system capacity and treatment guideline availability. Targets for AMS programmes were reported for both total care and the hospital sector, while targets for surveillance system capacity and availability of treatment guidelines were only reported for the total care sector. In general, indicator metrics were comparatively less specific compared with the other target categories, with professional requirements and work-time allocation for AMS programmes only specified for one target (Table 4).

## Discussion

We identified a total of 67 national AMC-related targets valid for 2025 or later, reported from 16 EU/EEA countries. Although our methodology is not fully comparable with previous studies, this is more than what was reported for the same countries in 2017 [7,8]. The influential role of the 2023 EU Council Recommendation through its recommended national AMC-related targets [1] was particularly evident in our findings. Nine EU countries reported having directly adopted, or being in the process of adopting, at least one of the two recommended national EU Council Recommendation targets in their NAPs or similar frameworks. In addition, several other countries stated they were considering aligning future updates of their NAPs with these recommended targets.

Our inventory can serve as a resource for policymakers and healthcare professionals seeking to mobilise action for prudent AMC, by providing examples of national targets and implementation frameworks used across a range of settings and resource levels. The AMC-related targets reported in our survey were broadly grouped into three categories: (i) population-level AMC targets, (ii) patient-level AMC targets and (iii) structural AMS targets. The feasibility for their implementation at national level will vary depending on national priorities, data availability and public health system organisation.

Population-level targets were by far the most commonly reported target category, likely reflecting their close alignment with current EU/EEA surveillance system capabilities. They are applicable even where national surveillance systems are less advanced, as monitoring can be done through the DDD information available from antimicrobial sales or reimbursement data sources. In addition, they can benefit from the standardised methodology and the benchmarking opportunities provided through ECDC ESAC-Net, as shown by several countries indicating the use of ESAC-Net data to set and monitor their national targets. However, while the broader population-level AMC targets may be sufficient for setting overarching goals, more specific targets focusing on particular patient groups or treatment indications might better guide prudent use. Only four countries reported having such patient-level AMC targets in place at national level. The data specificity required for this type of target typically includes detailed prescription information, which might not always be available at national level and may therefore limit wider adoption at present.

Our findings highlight the importance of robust and sustainable surveillance systems with well-functioning reporting mechanisms to support the development, monitoring and evaluation of national AMC targets. Systematically reviewing available data sources could allow for identification of additional indicators relevant for target setting and areas where significant gaps remain. Strengthening national digital public health infrastructures, particularly the integration of prescription data with electronic medical records and other health information systems, could improve the availability and completeness of data. This could further support the development of more context-specific AMC-related targets as well as facilitating the evaluation of progress in relation to changes in infectious disease morbidity, resistance trends and potential emergence of unintended consequences of changes in AMC policy and use [12].

Only a few structural AMS targets were reported in our survey, despite their importance in mobilising action towards prudent AMC [13,14]. The survey design and respondent profiles may have been less suited for capturing this target category, especially where indicators form part of broader infection prevention and control frameworks or are mainly assessed at local level through audits or point prevalence surveys. In addition, official health system requirements supporting prudent AMC, including specifications for AMS teams and surveillance infrastructures, might not be specifically perceived and monitored as targets. They could, however, benefit from having quantifiable components, as exemplified by the threshold target on minimum work-time requirements for AMS staff reported from Sweden. The low number of structural AMS targets reported from EU/EEA countries might also suggest that systematic monitoring of AMS capacity at national level is limited. Improving the availability and monitoring of AMS indicators could further support the development of definitions for AMS team composition and core activities [15], while embedding these indicators within national monitoring structures would strengthen accountability, ensure sustained resourcing and facilitate their integration into broader national AMC target frameworks.

While EU-level and national AMC-related targets can help mobilise national policymakers by providing clear directions and a structured framework for monitoring and evaluating progress, they might offer limited guidance for individual prescribers and local healthcare facilities. Feedback at regional and local levels, potentially supplemented with additional context-specific targets and benchmarking opportunities, could help operationalise these broader national goals and bridge the gap between high-level national policy and the necessary changes in local clinical practice. The healthcare workforce is a key lever for achieving AMC-related targets, and targeted training combined with supportive financial and organisational incentives can strengthen the adoption of practices aligned with national and local AMC guidelines [13,14]. While governments hold primary responsibility for ensuring prudent AMC, effective collaboration with regulators, healthcare providers, education bodies and community organisations remain essential [14].

Our survey targeted national ESAC-Net representatives with insight into national AMC-related strategies and achieved a high response rate from 25 of the 30 EU/EEA countries. The overview should, however, not be regarded as exhaustive, and some variation in the comparability and completeness of reported AMC-related targets is likely. The inventory may not reflect all elements of national policy, particularly where targets are embedded in complex governance structures, lie outside NAP frameworks, or are mainly monitored at regional or local levels. Differences in how countries define, implement, and oversee AMC and AMS indicators and targets may also influence comparability. In addition, the information in our inventory is reported as provided by the respondents; no systematic cross-checking against the cited references was performed, as these sources varied widely in language, format and accessibility.

With the continuous rise in AMR across the EU/EEA [16] and the limited progress towards the AMC-related targets set out in the EU Council Recommendation [17], reinforcing national action on prudent AMC remains an urgent public health priority. Besides clarifying strategic directions and providing a structure for monitoring and evaluating progress, AMC-related target frameworks can help clarify stakeholder roles, prioritise actions and guide cost estimates for implementation.

## Conclusions

Our survey showed that the majority of EU/EEA countries had established national AMC-related targets for 2025 or beyond, with the 2023 EU Council Recommendation appearing to be a key driver for the most recent target developments. Strengthening surveillance infrastructures remains critical for expanding target setting beyond general population-level AMC metrics, by facilitating increased use of patient-level AMC indicators and structural AMS indicators within national target frameworks. Regular progress reporting and introduction of supplementary and more context-specific targets at local and regional levels can further improve alignment between national policy objectives and clinical practice.

## Data Availability

All data generated or analysed during this study are included in this published article and the Supplementary material.

## ESAC-Net study group

Julia Weber: Federal Ministry of Labour, Social Affairs, Health, Care and Consumer Protection, Austria.

Lucy Catteau: Department of Epidemiology and public health, Sciensano, Belgium.

Arjana Tambic Andrasevic: University Hospital for Infectious Diseases and University of Zagreb School of Dental Medicine, Croatia.

Lenka Vostalová: Institute of Health Information and Statistics of Czechia, Czechiac.

Majda Attauabi: Statens Serum Institut, Denmark.

Janne Sepp: Estonian State Agency of Medicines, Estonia.

Emmi Sarvikivi: Finnish Institute for Health and Welfare (THL), Finland.

Ghaya Ben Hmidene: Santé Publique France, France.

Imke Wieters: Robert Koch Institute, Germany.

Lida Politi: National Public Health Organisation, Greece.

Ria Benko: University of Szeged, Faculty of Pharmacy, Institute of Clinical Pharmacy, University of Szeged, Albert-Szent-Györgyi Health Centre, Institute of Clinical Pharmacy, Hungary.

Anna Margrét Halldórsdóttir: Centre for Health Security and Communicable Disease Control, Directorate of Health, Iceland.

Marie Philbin: Antimicrobial Resistance and Infection Control, Health Services Executive Team, Health Services Executive, Ireland.

Filomena Fortinguerra: Italian Medicines Agency (AIFA), Italy.

Rolanda Valintėlienė: Institute of Hygiene, Lithuania.

Stéphanie Saleh: Health Directorate, Luxembourg.

Peter Zarb: Mater Dei Hospital, Ministry For Health, Malta.

Jan Baltink: Radboudumc, Netherlands.

Hege Salvesen: Blix Norwegian Institute of Public Health, Norway.

Aneta Mroczkowska: National Medicines Institute, Poland.

Joana Fernandes: INFARMED, I.P., Portugal.

Gabriel-Adrian Popescu: Matei Bals National Institute of Infectious Diseases, Romania.

Tomas Tesar: Faculty of Pharmacy, Comenius University, Slovakia.

Maja Subelj: National Institute of Public Health, Slovenia.

Ragda Obeid: The Public Health Agency of Sweden, Sweden.

## Supplementary Data

321000 Supplementary Material
