# Supplementary material for: Mapping nationally adopted targets for antimicrobial consumption and stewardship indicators in the human health domain, 25 European Union/European Economic Area countries, 2025
Source: Euro Surveill. 2026 Aug 27;31(34):2500976. doi: 10.2807/1560-7917.ES.2026.31.34.2500976 (PMC13525357; doi:10.2807/1560-7917.ES.2026.31.34.2500976)
Supplement: Supplementary file 1 [file 25-00976_HOGBERG_Supplement.pdf]

Supplementary material

This supplementary material is hosted by *Eurosurveillance* as supporting information alongside the article 'Mapping nationally adopted targets for antimicrobial consumption and stewardship indicators in the human health domain, 25 European Union/European Economic Area countries, 2025', on behalf of the authors, who remain responsible for the accuracy and appropriateness of the content. The same standards for ethics, copyright, attributions and permissions as for the article apply. Supplements are not edited by *Eurosurveillance* and the journal is not responsible for the maintenance of any links or email addresses provided therein.

Survey on national targets for human antimicrobial consumption/use in EU/EEA countries

Dear ESAC-Net colleagues,

The objective of this survey is to map national targets for human antimicrobial consumption/use and stewardship activities in EU/EEA countries. The survey is sent to the ESAC-Net target study group, including one person per country nominated by the National Focal Points for antimicrobial consumption earlier this year.

The results are planned to be discussed and presented at an ESAC-Net network webinar in June and in an article that we are drafting for submission to a peer-reviewed journal later this year.

We have tried to keep the survey as clear and concise as possible. If you have any questions, please do not hesitate to contact the coordinator of the survey.

We are looking forward to your answer to the same email addresses before 23 May 2025.
Many thanks for your contribution!
The ECDC ESAC-Net team

Respondent
Name:
E- mail address:
Country:

Has your country established national targets for human antimicrobial consumption/use?

This includes targets for reduction or optimisation of antimicrobial consumption/use, including targets related to proportion of certain World Health Organization (WHO) Access Watch Reserve (AWaRe) groups (if modified WHO AWaRe groups definitions are applied nationally, please describe), other antimicrobial groupings (e.g., broad/narrow spectrum antibiotics), or requirements for antimicrobial stewardship programmes/teams.

The targets can be focused on total consumption/use at national level or specific for certain health care settings/health specialities. They can apply to all or specific antimicrobials, or specific syndromes/infections. You will be able to specify this later.

☐ **No.** Are discussions being held or plans being developed to introduce national targets? If yes, are you able share any preliminary information? Enter your reply in the text box below.

☐ **Yes.** Please list the targets in the table on next page (add more rows if needed). Grey fields represent dummy examples to guide response structure.

Setting (community care, hospital care, etc) and geographical areas (national, regional) covered		Objective (Specific target or goal to achieve)	Antimicrobial groups covered	Unit of measure	Target	Year by which the target must be reached	Is the target part of a broader national or regional antimicrobial resistance strategy or action plan?<br><br>If yes, please briefly describe the strategy or action plan, and attach the complete strategy or provide link to it.	Is progress towards this target regularly monitored and reported?<br><br>If yes, please briefly describe to whom, how progress is measured (e.g. monitoring of national consumption data, audits of healthcare facilities etc.), how often and how it is reported. If progress is monitored and presented thorough official reports or dashboards, please provide links or copies of these.
Example	*Total care*<br>*National*	*Reduce total antimicrobial consumption.*	*ATC group J01*	*DDD per 1 000 inhabitants per day*	*By 20%*	*2030*	*Yes.*<br>*The target is part of a National Action Plan strategy (www.xxx.yy), which is aligned with the EU One Health Action Plan against Antimicrobial Resistance (AMR) and the WHO Global Action Plan on AMR.*	*Yes.*<br>*Healthcare facilities are mandated to regularly (annually) collect and evaluate data on antibiotic consumption. It supports local Antibiotic Stewardship programs and informs targeted interventions. Progress has to be reported to the MoH by June for the previous year. The report is publicly available on the web site www.xxx.yy*
Example	*Hospital care*<br>*National*	*Reduce consumption of “reserve antibiotics” (according to the national list of reserve antibiotics created by the National Health Insurance Fund).*	*See https:/ xxx.yy for definitions of national reserve antibiotics*	*DDD per 100 patient days*	*Below national median for 2020*	*2026*	*Yes.*<br>*The target is set by National Health Insurance Fund in document x, available at https:/ xxx.yy*	*Yes.*<br>*It is monitored by National Health Insurance fund on an annual basis. Results are mainly used internally and not regularly published in official reports.*
Example	*Community care*<br>*Region X*	*Increase proportion of patients treated for tonsillopharyngitis with penicillin V.*	*ATC group J01*	*% patients treated for tonsillopharyngitis with penicillin V/all antibiotic prescriptions for tonsillopharyngitis*	*> 90%*	*2027*	*No. It was introduced by the regional society for p rudent use of antimicrobials.*	*Yes, annually on the website of the regional society for prudent use of antimicrobials www.xxx.yy.*

Survey replies

Country-specific information from the survey and related communication is summarised below.

Austria
Austria did not report any AMC-related targets but has ongoing and formalised monitoring of several antimicrobial stewardship indicators as part of the national quality standard for antimicrobial use in hospitals [1]. These indicators monitor the implementation of nationwide recommendations for antimicrobial stewardship teams and related activities, serving as a tool to support action for physicians, hospital management, specialist pharmacists and hospital operators. Although no quantifiable target values have been set for these indicators, their status will be evaluated for a subset of hospitals in 2029.

Belgium
The recently expired Belgian One Health National Action Plan on the Fight against Antimicrobial Resistance 2020–2024 included several AMC-related targets for both the community and hospital sectors [2]. Although the target endpoint years (2019 and 2024) have passed, the associated indicators continue to be monitored outside of the target framework. A new national action plan is currently under preparation and is expected to establish new AMC-related targets for coming years.

Beyond the AMC-related target framework linked to past and upcoming national action plans, additional targets have been established through other national initiatives. These targets are not set for specific years but establish thresholds for desired maximum or minimum levels of AMC-related indicators for the community and hospital sectors separately.

For antibiotics prescribed by general practitioners and reimbursed by the national health insurance, the National Institute for Health and Disability Insurance (NIHDI) introduced four continuous AMC-related targets in 2023 [3]. These targets are approved by the Conseil national de la promotion de la qualité (CNPQ) and published in a Royal Decree, and set thresholds for a maximum of 23% of adults patients (≥15 years) and 45% of paediatric patients (<15 years) receiving an antibiotic prescription (ATC J01) among all patients with a reimbursed pharmaceutical prescription; at least 80% amoxicillin (ATC J01CA04) out of the total defined daily doses (DDD) for amoxicillin (ATC J01CA04) and amoxicillin with beta-lactamase inhibitor (ATC J01CR02) consumption combined; and a maximum of 20% second-line antibiotics (amoxicillin combined with clavulanic acid ATC J01CR02, cephalosporins ATC J01D, quinolones ATC J01M and macrolides ATC J01FA) out of the total DDD for antibacterials for systemic use (ATC J01). Compliance with these is evaluated annually by NIHDI at the level of individual prescribers, who receive personalised feedback and potential corrective measures. Although not yet part of a national AMC monitoring system, these indicators represent a recent national strategy to support prudent antibiotic prescribing in primary care.

At the hospital level, the establishment of an antibiotic stewardship team is mandated by Royal Decree in all general hospitals with at least 150 beds since 2008 [4]. The decree defines the minimum team composition and core responsibilities; however, no full-time equivalents are specified for any team member. As such, while the structure is legally required, its implementation may vary across hospitals. Although hospitals are required to submit an annual report to the Belgian Antibiotic Policy Coordination Commission (BAPCOC), national analyses of these reports were only conducted up to 2011, and no structured national monitoring or feedback to individual stewardship teams has been organized since. This lack of systematic oversight was identified as a limitation in the Belgian antimicrobial stewardship infrastructure in several national and international evaluations and was included as an action point in the Belgian action plan 2020–2024. In response, BAPCOC funded the Belgian Evaluation of Antimicrobial Stewardship Teams (BEAST) project, coordinated by Sciensano with support from Belgian and international experts, to reassess the functioning, needs, and resources of hospital antimicrobial stewardship teams.

Croatia
Croatia reported that although reduced AMC is included as an indicator in the current AMR national

action plan, no target values are defined. The survey response indicated the potential for inclusion of quantified targets in future updates of the National Programme for the Control of Antibiotic-Resistant Bacteria. Several quality indicators for rational antibiotic prescribing have been established by the Croatian Health Insurance Fund, which provides financial incentives to hospitals and physicians to discourage the use of reserve or high-cost antibiotics. The definitions of these quality indicators are currently under review.

Czechia
No AMC-related targets were reported from Czechia. However, the Strategy of the National Antibiotic Programme of the Czech Republic for the period 2025–2031, currently under development, is expected to define goals for reducing antibiotic consumption in line with international recommendations.

Denmark
Denmark did not report any AMC-related targets but has ongoing and formalised monitoring of several AMC-related indicators as outlined in the Danish National Action Plan for Antimicrobial Resistance in Humans, published by the Danish Ministry of Health in 2025 [5]. These indicators cover both total antibiotic consumption and consumption of specific antimicrobial groups in the community and hospital sectors. The action plan includes a framework for monitoring and evaluating trends in these indicators, but does not specify any quantifiable or time-bound targets.

Estonia
No AMC-related targets were reported from Estonia. However, the Estonian National Antimicrobial Resistance Action Plan for Human Health 2026-2030, currently under development to support the recently published Estonian Antimicrobial Resistance Management Strategy 2025-2030 [6] is expected to set national targets related to reductions in antimicrobial consumption.

Finland
The Finnish national antimicrobial resistance action plan 2025-2028 [7] includes two targets based on the EU Council Recommendation [8]; a reduction of total consumption of antibiotics for systemic use (ATC J01) by 9% in 2030 compared with the baseline year 2019 and ensuring that at least 65% of the WHO AWaRe antimicrobials belongs to Access group by 2030. Current priorities focus on strengthening the existing AMC surveillance system to increase data granularity to better inform targeted stewardship actions.

France
The French National Strategy on Prevention of Infections and Antibiotic Resistance (2022–2025) [9] lists several AMC-related targets to be achieved by 2025, with a recent extension of the targets' end year to 2027. Besides an overarching national target of having fewer than 20 DDD per 1000 inhabitants per day for antibacterials for systemic use (ATC J01) consumed in all healthcare sectors combined by 2027, there are also several separate targets for the community, hospital sector and long-term care facilities. Some targets are also further limited to specific patient groups or diagnostic criteria.

For the community sector, the 2027 targets include fewer than 650 prescriptions of antibacterials for systemic use (ATC J01) per 1000 inhabitants per year; fewer than 20 prescriptions of antibacterials for systemic use (ATC J01) per 100 patients aged 16-65 years attending a physician and with no long-term illness per year; and at least a 20% decrease in consumption of the critical antibiotics for systemic use defined by the Société de Pathologie Infectieuse de Langue Française (SPILF) [10] compared with 2019 and measured as DDD per 1000 inhabitants per day.

For the hospital sector, the 2027 targets include at least a 10% decrease in consumption of antibacterials for systemic use (ATC J01), rifampicin (ATC J04AB02), oral imidazole (ATC P01AB) and fidaxomicin (ATC A07AA12) compared with 2019 and measured as DDD dispensed in hospitals to hospitalised patients per 1000 hospital days; at least a 10% reduction in the proportion of broad-spectrum antibiotics (third- and fourth generation cephalosporins, piperacillin-tazobactam, aztreonam, carbapenems, fluoroquinolones, glycopeptides, linezolid, tedizolid, daptomycin and colistin) out of

total hospital consumption of antibacterials for systemic use (ATC J01) compared with 2019; and at least 80% of antibiotic prescriptions for lower respiratory tract infections having a treatment duration of fewer than seven days measured as proportion of compliant health records out of total records.

For long-term care facilities, the 2027 target is at least a 20% reduction in consumption of the critical antibiotics for systemic use defined by SPILF [10], dispensed by community pharmacies and in-house pharmacies for residents in long-term care facilities compared with 2019 and measured as DDD per 1000 long-term care facilities residents per year or DDD per 1000 hospital days.

The national strategy also provides a framework specifying stakeholder responsibility by detailing data sources, geographical granularity, and frequency of reporting (annual to biannual). The latest progress towards the targets was published by the French National Public Health Agency in 2024 [11].

Germany
Two nationally adopted AMC-related targets are listed in the Action Plan 2024–2026 [12] of the German Antimicrobial Resistance Strategy (DART 2030) [13]. These targets reflect those suggested for Germany in the EU Council Recommendation, and include a 9% reduction in total consumption of antibacterials for systemic use (ATC J01) between 2019 and 2030 as well as ensuring that at least 65% of the WHO AWaRe antimicrobials belong to the Access group by 2030. Both targets are based on data measured as DDD per 1000 inhabitants per day.

Progress towards these targets will be reviewed as part of the national action plan evaluation in 2026.

Greece
Greece's national AMR policy is currently transitioning from having no formal AMC-related targets to a structured, measurable, and integrated One Health framework. The new national Antimicrobial Resistance Action Plan for 2025–2030, currently under development and not yet published, introduces measurable national targets and structured monitoring mechanisms in line with those suggested for Greece in the EU Council Recommendation [8]. These include a 27% reduction in total consumption of antibacterial for systemic use (ATC J01) between 2019 and 2030 (measured as DDD per 1000 inhabitants per day) and at least 65% of the WHO AWaRe antimicrobials belonging to Access group by 2030.

Hungary
No national AMC-related targets were reported from Hungary. However, the national One-Health strategy to combat Antimicrobial Resistance, currently under approval, is expected to include indicators promoting the prudent use of antimicrobials.

Iceland
The Icelandic National Action Plan on Antimicrobial Resistance 2025–2029 [14], published by the Ministry of Health in 2024, lists several national AMC-related targets to be achieved by 2029. The action plan also provides an implementation plan, including details on stakeholder responsibility, priorities, and cost estimates.

For all healthcare sectors combined, the 2029 targets include total consumption of antibacterials for systemic use (ATC J01) not exceeding 15 DDD per 1000 inhabitants per day, and the WHO AWaRe antimicrobials in the Access group exceeding 80 % of the WHO AWaRe antimicrobials. Progress towards both targets is measured through sales data.

For the community sector specifically, the 2029 targets include a maximum consumption of antibacterials for systemic use (ATC J01), measured through prescription data, of 14 DDD per 1000 inhabitants per day; with tetracycline (ATC J01A) consumption not exceeding 2.5 DDD per 1000 inhabitants per day.

For the hospital sector, the 2029 targets specify a maximum consumption of antibacterials for systemic use (ATC J01), measured through sales data, of 1.0 DDD per 1000 inhabitants per day; with

third-generation cephalosporin consumption (ATC J01DD) not exceeding 0.15 DDD per 1000 inhabitants per day.

Progress towards these targets is monitored annually through a national report on antibiotic use and antibiotic susceptibility of bacteria in humans and animals, with the latest update dating from 2024 [15]. The national action plan working group also has the assignment to annually monitor the progress of the plan.

In addition to the antimicrobial consumption-specific targets, the national action plan also includes targets for related process indicators. These include ensuring that more than 90% of all antibiotic prescriptions in humans are recorded in the central database; that specified guidelines for targeted and prudent use of antibiotics are available for more than 95% of defined target groups (larger hospitals, nursing homes, primary care centres, and high prescribing specialists outside hospitals); and that active antibiotic stewardship programmes are implemented in more than 95% of health institutions by 2029. A Monitoring and Evaluation plan was published in 2025 [16]:

Ireland

The Irish Health Service Antimicrobial Resistance and Infection Control Action Plan 2022-2025 [17] which aligns with the Irish National Action Plan on AMR (iNAP2) [18], lists AMC-related targets for both the community and hospital sectors. The targets are to be achieved by the end of 2025 and include both consumption and process indicators derived from multiple data sources.

For the community sector, these 2025 targets include total consumption of antibacterials for systemic use (ATC J01) of fewer than 20.5 DDD per 1000 inhabitants per day (based on wholesale to community pharmacy sales data), and fewer than 101 antibiotic prescriptions/100 medical card patients (based on reimbursement data). In addition, fewer than 26% of a modified list of WHO AWaRe antimicrobial agents prescribed should belong to the Watch and Reserve groups. Community sector targets are monitored quarterly and reported to the Department of Health [19], with additional prescription-related metrics provided to each individual general practitioner allowing comparison of their own rates to national rates.

For the hospital sector, the 2025 targets include a maximum consumption of antibacterials for systemic use (ATC J01) of 72.1 DDD per 100 bed-days used (measured from pharmacy dispensing data) and a maximum of 20% of surgical antibiotic prophylaxis prescriptions extending beyond 24 hours (measured through the annual national point prevalence survey). The hospital targets are monitored annually and reported to the Department of Health and other relevant stakeholders [19-20].

Italy

The Italian National Action Plan to combat Antimicrobial Resistance 2022-2025 [21] lists several national and regional AMC-related targets to be achieved by 2025 in the community and hospital healthcare sectors.

For the community sector, the targets include a reduction of at least 10% in the overall consumption of antibacterials for systemic use (ATC J01); and a reduction of at least 20% in the secondary indicator for antimicrobial consumption in the community sector established by the ECDC, European Food Safety Authority (EFSA) and European Medicine Agency (EMA) (the ratio of consumption of mainly 'broad-spectrum' penicillins (ATC J01CR), 'broad-spectrum' cephalosporins (ATC J01DC and J01DD), macrolides (ATC J01FA) except erythromycin (ATC J01FA01)) and fluoroquinolones (ATC J01MA) to the consumption of narrow-spectrum penicillins (ATC J01CA, J01CE and J01CF), narrow-spectrum cephalosporins (ATC J01DB) and erythromycin (ATC J01FA01) [22]. Both targets are based on the DDD per 1000 inhabitants per day metric. In addition, three national community targets specifically address antimicrobial use among children aged 0 and 13 years, measured as the number of packages per 1000 inhabitants per year. These include at least 10% reduction in total consumption of antibacterials for systemic use (ATC J01); at least 30% increase of the ratio of consumption of amoxicillin (ATC J01CA04) to amoxicillin/clavulanic acid (ATC J01CR02); and at least 20% reduction of the ECDC, EFSA and EMA secondary indicator for the community sector [22].

For the hospital sector, targets include at least a 5% reduction in overall consumption of antibacterials for systemic use (ATC J01) measured as DDD per 100 hospitalisation days, and at least a 10% reduction in the consumption of fluoroquinolones (ATC J01MA) and carbapenems (ATC J01DH) respectively.

In addition to the targets listed in the action plan, the Italian Medicines Agency (AIFA) introduced two additional targets in their reporting framework in 2020 [23]. These threshold targets do not have a defined end year. The first stipulates that at least 65% of antibacterials should belong to the Access group according to the WHO AWaRe classification; and the second requires that the ECDC, EFSA, EMA secondary indicator for hospital antimicrobial consumption (proportion of glycopeptides (ATC J01XA), third- and fourth-generation cephalosporins (ATC J01DD and J01DE), monobactams (ATC J01DF), carbapenems (ATC J01DH), fluoroquinolones (ATC J01MA), polymyxins (ATC J01XB), piperacillin and enzyme inhibitor (ATC J01CR05), linezolid (ATC J01XX08), tedizolid (ATC J01XX11), and daptomycin (ATC J01XX09) out of total hospital consumption of antibacterials for systemic use (ATC J01)) [22] remains the same or below the level of the population-weighted EU/EEA mean reported by ECDC ESAC-Net.

Progress towards all targets is monitored through the national AMC surveillance systems and reported annually on the AIFA website. The latest report available refers to data from 2023 [24].

Lithuania
The Implementation Plan of the Programme of the 19th Government of the Republic of Lithuania includes an AMC-related structure and process target aiming to establish antimicrobial stewardship programmes in at least 85% of Lithuanian hospitals by 2028 [25]. Progress towards this target is primarily monitored internally and not regularly published in official reports. Additional AMC-related targets are currently under discussion for inclusion in the updated Lithuanian antimicrobial resistance action plan planned for the period 2026-2027.

Luxembourg
Luxembourg is in a transition phase following the expiration of the first National Antibiotics Plan One Health 2018–2022 (extended until 2024) [26], with the new national action plan under preparation and expected to be published in early 2026. While the first action plan did not include quantifiable AMC-related targets, the forthcoming plan is expected to include two targets for 2030 aligned with those suggested for Luxembourg in the EU Council Recommendation [8]. These targets include an 18% reduction in consumption of antibacterials for systemic use (ATC J01) compared with 2019 levels and ensuring that at least 65% of antibiotics belong to the Access group according to the WHO AWaRe classification.

Progress towards these targets will be monitored through the annual data collection by the Health Directorate and reported in its annual national report monitoring antibiotic consumption, antibiotic resistance and the presence of antibiotic residues in Luxembourg [27].

Malta
Three AMC-related targets to be achieved by 2030 were reported from Malta, all developed to support the Strategy and Action Plan for the Prevention and Containment of Antimicrobial Resistance in Malta (2020–2028) [28].

For all healthcare sectors combined, the 2030 targets include an 18% reduction in the consumption of antibacterials for systemic use (ATC J01) compared with 2019 and ensuring that at least 65% of antimicrobials belong to the Access group according to the WHO AWaRe classification. These objectives are consistent with the targets proposed for Malta in the EU Council Recommendation.

For the hospital sector, the 2030 target is to maintain the ECDC, EFSA, EMA secondary indicator for the hospital sector antimicrobial consumption [22] below the population-weighted EU/EEA mean as reported by ECDC ESAC-Net.

All targets are based on consumption measured as DDD per 1000 inhabitants per day and are monitored through reports from the National Antibiotic Committee, with the latest available report from 2022 [29].

The Netherlands
The Netherlands' National Action Plan for the Reduction of Antimicrobial Resistance 2024–2030 [30] defines two national targets aligned with those proposed for the country in the EU Council Recommendation. These include a minimum 3% reduction in the total consumption of antibacterials for systemic use (ATC J01) by 2030 compared with 2019 and at least 65% WHO AWaRe antimicrobials belonging to the Access group by 2030.

The action plan is scheduled to be evaluated by the Ministry of Health, Welfare and Sport, Ministry of Agriculture, Fisheries, Food Security and Nature and Ministry of Infrastructure and Water Management every three years, with the first evaluation planned for 2027.

Norway
The Norwegian National One Health Strategy Against Antimicrobial Resistance 2024–2033, issued in 2024, sets a target of a 10% reduction in the total use of antibacterials for systemic use (ATC J01) excluding methenamine (ATC J01XX05) by 2033 compared with 2019 levels [31].

The previous national action plan included several sector-specific goals for the period 2015-2020, including reductions in overall consumption; consumption of antibiotics for respiratory tract infections in the community; and of broad-spectrum antibiotics in hospitals [32]. The end point of one of these expired targets, i.e. to limit consumption of antibacterials for systemic use (ATC J01) in the community sector to a maximum of 250 prescriptions per 1000 inhabitants per year, is still being used as a threshold target towards which consumption data are regularly monitored. Work on an updated national action plan to support the 2024–2033 strategy is under way and is expected to include additional AMC-related targets and indicators.

Progress towards targets is monitored at the national level through the national AMC surveillance system and reported annually in the NORM/NORM-Vet reports [33].

Poland
Poland did not report any AMC-related targets. However, a national action plan is currently under development and is expected to be aligned with the recommended targets for Poland in the EU Council Recommendation.

Portugal
The Updates to the Infection Prevention and Control and Antimicrobial Resistance Program (PPCIRA), published by the Secretary of State for Health Promotion Office in 2022 [34] and amended in 2023 [35] define several AMC-related targets. These targets do not have a specified end year, and either specify annual reductions relative to the previous year or set thresholds prescribing maximum levels of consumption of specific antimicrobial groups. They are integrated within the broader national patient safety and public health strategies and are continuously monitored and adjusted by the PPCIRA as the coordinating body.

For the community sector, targets include reductions of consumption of antibacterials for systemic use (ATC J01); and the ECDC, EFSA, EMA secondary indicator for the community sector compared with the previous year.

For the hospital sector, target thresholds are set to fewer than 5000 DDD of antibacterials for systemic use (ATC J01) per 1000 patient discharges or at least a 20% reduction compared with the previous year, and fewer than 200 DDD of carbapenems (ATC J01DH) per 1000 patient discharges or at least 20% reduction compared with the previous year. These thresholds serve as alert indicators rather than absolute targets, triggering PPCIRA to respond by developing an improvement plan. The indicators are interpreted together with supplementary information on infection rates, stewardship interventions and clinical appropriateness of prescriptions.

Progress is reported quarterly at regional and local healthcare institution levels and annually at national level [36]. Data on hospital care indicators (in terms of DDD and units consumed) are also available through the National Authority of Medicines and Health Products (INFARMED, I.P.) antibiotics dashboard [37].

Romania
No nationally adopted AMC-related targets were reported from Romania.

Slovakia
Slovakia's new national antimicrobial resistance action plan 2025 - 2030, currently under development and not yet published, will introduce measurable national targets and structured monitoring mechanisms. The targets are based on the EU Council Recommendation and include a reduction of total consumption of antibiotics for systemic use (ATC J01) by 9% in 2030 compared with the baseline year 2019, and ensuring that at least 65% of the WHO AWaRe antimicrobials belongs to Access group by 2030. Progress will be monitored through the data reported to ECDC ESAC-Net [38].

Slovenia
In Slovenia, the recently expired National One Health Strategy for the Management of Microbial Resistance 2019–2024 [39] included several AMC-related targets, aimed at reducing overall consumption and optimising prescribing practices. Although the formal target period ended in 2024, the associated indicators covering both various aspects of antimicrobial consumption as well as guideline-based prescribing audits continue to be monitored outside the target framework. Monitoring is coordinated by the National Institute of Public Health (NIJZ) and reported annually to the Ministry of Health and other relevant stakeholders. As of 2025, Slovenia has not publicly adopted a successor national target framework for antimicrobial consumption or stewardship; the monitoring and stewardship structures remain in place without a formally published road map for the period 2025 and beyond.

Sweden
The Swedish Strategy to Combat Antibiotic Resistance 2026-2035 [40] includes three AMC-related targets to be achieved by 2030 and 2035. The targets for total consumption include a reduction of total consumption of antibiotics for systemic use (ATC J01) by 3% in 2030 compared with the baseline year 2019 and ensuring that the proportion of WHO AWaRe antimicrobials in the Access group remain above 75% by 2035. While the first target is harmonised with the target suggested for Sweden by the EU Council Recommendation, the second target is higher compared to both the 65% WHO AWaRe Access group threshold recommended by the European Council [8] and the 70% threshold included in the United Nations Political Declaration of the High-level Meeting on Antimicrobial Resistance published in 2024 [41]. Finally, for the community sector, the strategy sets a target of a maximum of 250 prescriptions per 1000 inhabitants per year for antibacterials for systemic use (ATC J01) excluding methenamine (ATC J01XX05) nationally and in all Swedish regions by 2035, based on recommendations by the Swedish Strategic Programme against Antibiotic Resistance (Strama) [42]. In addition to the targets introduced in the Swedish strategy for 2026-2035, Strama recommends several additional AMC-related indicators and targets, covering both the community [42] and hospital sectors [43]. These targets are not set for specific years but establish thresholds for desired maximum or minimum levels of consumption.

For the community sector, the Strama targets for antibiotic use in ambulatory care recommends that for children aged 0-6 years, phenoxymethylpenicillin (ATC J01CE02) should constitute at least 80% of the antibiotics commonly prescribed for respiratory tract infections (including penicillin ATC J01CE02, amoxicillin ATC J01CA04, amoxicillin with enzyme inhibitor ATC J01CR02, cephalosporins ATC J01DB-DE and macrolides ATC J01FA). For women aged 18–79 years, ciprofloxacin (ATC J01MA02) and norfloxacin (ATC J01MA06) should represent a maximum of 10% antibiotics commonly prescribed to treat urinary tract infections (UTI) (including pivmecillinam ATC J01CA08, trimethoprim ATC J01EA01, ciprofloxacin ATC J01MA02, norfloxacin J01MA06 and nitrofurantoin J01XE01) [42]. Adherence to these targets is monitored through the national Swedish surveillance system for antimicrobial sales, and reported nationally and regionally on a monthly basis through an interactive fact sheet compiled

by the Public Health Agency of Sweden [44], as well as on an annual basis by the Public Health Agency of Sweden through the national report on sales of antibiotics and occurrence of antibiotic resistance in Sweden [45].

At hospital level, the Strama national inpatient indicators for antibiotics provides a framework of multiple indicators and data sources supporting diagnosis-linked and organisational monitoring of stewardship efforts in inpatient settings [43]. While establishment of locally adapted targets is recommended for most of these indicators, the document recommends a maximum of 10% and 20% fluoroquinolones (ATC J01MA) use in women and men, respectively, out of the antibiotics used to treat afebrile UTI in inpatients (fluoroquinolones ATC J01MA, pivmecillinam ATC J01CA08, nitrofurantoin ATC J01XE01 and trimethoprim ATC J01EA01). These hospital targets are monitored at the local level through electronic tools to support local quality improvement efforts. The coverage of these tools varies between regions and care settings.

In addition, Strama is also proposing a target for hospitals to have personnel dedicated to antibiotic stewardship work of at least 0.1 full-time equivalent per 100 beds [43]. Progress towards this target is not systematically monitored.

References

1. Bundesministerium für Arbeit, Soziales, Gesundheit, Pflege und Konsumentenschutz (BMSGPK) [The Federal Ministry of Labour, Social Affairs, Health, Care and Consumer protection]. Qualitätsstandard Antiinfektiva – Anwendung in Krankenanstalten. Antimicrobial Stewardship [Quality standard for anti-infectives - Application in hospitals. Antimicrobial stewardship]. Vienna:BMSGPK;2024. German. Available at: https://www.sozialministerium.gv.at/Themen/Gesundheit/Gesundheitssystem/Gesundheitssystem-und-Qualitaetssicherung/Qualitaetsstandards/Qualitaetsstandard-Antiinfektiva-Anwendung-in-Krankenanstalten.html
2. Federal Public Service (FPS) Health, Food Chain Safety and Environment. Belgian One Health National Action Plan on the fight against antimicrobial resistance 2020-2024. Brussels:FPS; 2021. Available at: https://www.health.belgium.be/sites/default/files/uploads/fields/fpshealth_theme_file/en-amr_one_health_national_plan_final_1.pdf
3. Institut national d'assurance maladie-invalidité [National Institute for Health and Disability Insurance]. Antibiotiques: indicateurs pour une prescription efficace par les médecins généralistes [Antibiotics: indicators for effective prescribing by general practitioners]. French. Website, accessed 13 November 2025. Available from : https://www.inami.fgov.be/fr/themes/qualite-des-soins/indicateurs/antibiotiques-indicateurs-pour-une-prescription-efficace-par-les-medecins-generalistes
4. Service public fédéral Santé publique, Sécurité de la Chaîne alimentaire et Environnement [Federal Public Service Health]. Arrêté royal modifiant l'arrêté royal du 4 mars 1991 fixant les normes auxquelles une officine hospitalière doit satisfaire pour être agréée [Royal Decree amending the Royal Decree of March 4, 1991 setting the standards that a hospital pharmacy must meet in order to be approved]. 2008. Available from: https://www.ejustice.just.fgov.be/cgi/article_body.pl?language=fr&caller=summary&pub_date=08-03-28&numac=2008024120
5. Ministry of the Interior and Health of Denmark (ISM). National action plan on antimicrobial resistance in humans. Copenhagen:ISM;2025. Available at: https://www.ism.dk/Media/638918721595453894/National-action-plan-on-antimicrobial-resistance-in-humans-UK-TILG.pdf
6. Sotsiaalministeerium [Ministry of Social Affairs] Eesti antimikroobse resistentsuse ohjamise strateegia 2025–2030 (Antimicrobial Resistance (AMR) management strategy 2025–2030). Tallin: Sotsiaalministeerium; 2024. Estonian. Available from: https://www.sm.ee/sites/default/files/documents/2024-11/Eesti%20AMR%20ohjamise%20strateegia_28.11.pdf
7. Finnish Institute for Health and Welfare. Mikrobilääkeresistenssin torjunnan kansallinen toimintaohjelma 2025-2028. Toimeenpano-ohjelma, vuoden 2025 suunnitelma [National Action Plan for Antimicrobial Resistance, Implementation plan for the years 2025-2028 and plan for the year 2025]. Helsiniki: Finnish Institute for Health and Welfare;2025. Finish. Available from: https://www.julkari.fi/handle/10024/151957
8. Council of the European Union. Council Recommendation on stepping up EU actions to combat antimicrobial resistance in a One Health approach (2023/C 220/01). Official Journal of the European Union C 220/1. Available at: https://eurlex.europa.eu/legal-1content/EN/TXT/?uri=CELEX:32023H0622(01)
9. French Ministry of Solidarity and Health. National strategy for preventing infections and antibiotic resistance 2022–2025. Paris: French Ministry of Solidarity and Health;2022. Available at : https://sante.gouv.fr/IMG/pdf/national_strategy_for_preventing_infections_and_antibiotic_resistance_2022-2025_.pdf
10. Société de Pathologie Infectieuse de Langue Française (SPILF) [French-Speaking Society of Infectious Pathology]. Actualisation de la liste des antibiotiques critiques disponibles en France pour l'exercice libéral et en établissements de santé [Update of the list of critical antibiotics available in France for private practice and in healthcare establishments]. Paris: SPILF;2022.French. Available at:

https://www.infectiologie.com/UserFiles/File/spilf/recos/saisine-dgs-atb-critique-9-fev-22-v3.pdf
11. Santé publique France (SpF) [French National Public Health Agency]. Prévention de la résistance aux antibiotiques: une démarche "Une seule santé". [Preventing Antibiotic Resistance: A "One Health" Approach]. Paris:SpF;2024. French. Available at: https://www.santepubliquefrance.fr/maladies-et-traumatismes/infections-associees-aux-soins-et-resistance-aux-antibiotiques/resistance-aux-antibiotiques/documents/rapport-synthese/prevention-de-la-resistance-aux-antibiotiques-une-demarche-une-seule-sante-.-novembre-2024
12. Bundesministerium für Gesundheit (BMG) [Federal Ministry of Health] Aktionsplan zur DART 2030, 2024-2026 [Action plan for DART 2030, 2024-2026]. Berlin: BMG; 2024. German. Available at: https://www.bundesgesundheitsministerium.de/fileadmin/Dateien/3_Downloads/A/Antibiotika-Resistenz-Strategie/240510_1._Aktionsplan_zur_DART_2030.pdf
13. Bundesministerium für Gesundheit (BMG) [Federal Ministry of Health]. DART 2030: German Antimicrobial Resistance Strategy. Berlin:BMG;2023. Available at: https://www.bundesgesundheitsministerium.de/fileadmin/Dateien/3_Downloads/A/Antibiotika-Resistenz-Strategie/DART_2030_en.pdf
14. Ministry of Health (MoH). National Action Plan (NAP) on Antimicrobial Resistance 2025-2029. Reykjavik:MoH;2024. Available at: https://www.stjornarradid.is/library/04-Raduneytin/Heilbrigdisraduneytid/ymsar-skrar/A%c3%b0ger%c3%b0a%c3%a1%c3%a6tlun%20gegn%20s%c3%bdklalyfja%c3%b3n%c3%a6mi%20EN.pdf
15. Embætti landlæknis [The Directorate of Health]. Sýklalyfjanotkun og sýklalyfjanæmi baktería hjá mönnum og dýrum á Íslandi 2024 [Antibiotic use and antibiotic susceptibility of bacteria in humans and animals in Iceland 2024]. Reykjavik:Embætti landlæknis;2025. Icelandic. Available at: https://island.is/syklalyfjaanaemi-og-syklalyfjanotkun/skyrslur
16. Ministry of Health (MoH). National Action Plan on Antimicrobial Resistance 2025-2029. Monitoring and Evaluation. Reykjavik: MoH;2025. Available at: https://www.stjornarradid.is/library/04-Raduneytin/Heilbrigdisraduneytid/ymsar-skrar/250423%20Heilbrigdisraduneytid%20Syklalyfjaonaemi%202025%20EN%20V4.pdf
17. Health Service Executive (HSE) HSE antimicrobial resistance and infection control action plan 2022-2025. Dublin:HSE;2021. Available from : https://www.hse.ie/eng/about/who/healthwellbeing/our-priority-programmes/hcai/resources/general/hse-antimicrobial-resistance-infection-control-amric-action-plan-2022-2025.pdf
18. Government of Ireland. Ireland’s One Health National Action Plan on Antimicrobial Resistance 2021 – 2025 (iNAP2). Dublin, Government of Ireland; 2021. Available from : https://www.gov.ie/en/publication/d72f1-joint-action-on-antimicrobial-resistance/
19. Health Service Executive, Health Protection Surveillance Centre. Surveillance reports. Antibiotic consumption. Website. https://www.hpsc.ie/a-z/microbiologyantimicrobialresistance/europeansurveillanceofantimicrobialconsumptionesac/
20. Health Service Executive (HSE). Report on Antimicrobial Point Prevalence Survey in Acute Hospitals in Ireland 2024 Surgical Antibiotic Prophylaxis Report. Dublin: HSE;2025. Available at: https://www.hse.ie/eng/services/list/2/gp/antibiotic-prescribing/hospital-related-guidelines/national-antimicrobial-pps-2024-surgical-antibiotic-prophylaxis-report.pdf
21. Ministero della Salute [Ministry of Health]. Piano Nazionale di Contrasto all’Antibiotico-Resistenza (PNCAR) 2022-2025 [National Plan to Combat Antibiotic Resistance (PNCAR) 2022-2025]. Rome:Ministero della Salute;2022. Italian. Available at: https://www.salute.gov.it/imgs/C_17_pubblicazioni_3294_allegato.pdf
22. European Centre for Disease Prevention and Control (ECDC), European Food Safety Authority (EFSA) Panel on Biological Hazards and European Medicines Agency (EMA) Committee for Medicinal Products for Veterinary Use. ECDC, EFSA and EMA Joint Scientific Opinion on a list of outcome indicators as regards surveillance of antimicrobial resistance and antimicrobial consumption in humans and food-producing animals. EFSA Journal 2018;15(10):5017. Available at: www.ecdc.europa.eu/sites/default/files/documents/AMR-indicators-joint-report-Oct-2017.pdf

23. The Medicines Utilisation Monitoring Centre. National Report on antibiotics use in Italy. Year 2020. Rome: Italian Medicines Agency, 2022. Available at: https://www.aifa.gov.it/documents/20142/1664282/Rapporto_Antibiotici_2020.pdf
24. The Medicines Utilisation Monitoring Centre. National Report on antibiotics use in Italy. Year 2024. Rome: Italian Medicines Agency, 2026. Available from: https://www.aifa.gov.it/documents/20142/3575501/Rapporto_Antibiotici_2024.pdf
25. Government of the Republic of Lithuania. Devynioliktosios Lietuvos Respublikos Vyriausybės Programos Nuostatų Igyvendinimo Planas [Implementation plan of the program provisions of the nineteenth government of Lithuania.] Lithuanian. Available from: https://e-seimas.lrs.lt/portal/legalAct/lt/TAD/12f5cd91062911f0a2ad91db29a9514e?jfwid=57lm4w4h5
26. Ministère de l'Agriculture, de la Viticulture et du Développement rural, Ministère de la Santé [Ministry of Agriculture, Viticulture and Rural Development, Ministry of Health] Antibiotiques - Plan National Antibiotiques One Health 2018-2022 (prolongé jusqu'en 2024) [National Antibiotics Plan One Health 2018-2022 (extended until 2024)]. Luxembourg:Gouvernement du Grand-Duché de Luxembourg;2018. French. Available at : https://santesecu.public.lu/fr/publications/p/plan-national-antibiotiques-2018-2022.html
27. Direction de la Santé [Health Directorate] Plan National Antibiotiques (2025). Surveillance de la consommation d'antibiotiques, de l'antibiorésistance et de la présence de résidus d'antibiotiques au Luxembourg. Luxembourg:Direction de la Santé:2025. Available from: Rapport (Sept 2025): Surveillance de la consommation d'antibiotiques, de l'antibiorésistance et de la présence de résidus d'antibiotiques au Luxembourg - Portail Santé - Luxembourg
28. Ministry for health, Ministry for agriculture, fisheries & animal rights. A Strategy and Action Plan for the Prevention and Containment of Antimicrobial Resistance in Malta (2020 – 2028) 2020. Valetta;2020. Available at: https://antibiotics.gov.mt/wp-content/uploads/2025/03/AMR-Strategy-Final-Jul-2020.pdf
29. National Antibiotic Committee Malta. Antimicrobial Consumption and Resistance in Malta. Annual report 2022. Available at: https://antibiotics.gov.mt/wp-content/uploads/2024/04/National-Antibiotic-Committee-2021-report.pdf
30. Ministerie van Volksgezondheid, Welzijn en Sport, Ministerie van Landbouw, Natuur en Voedselkwaliteit, Ministerie van Infrastructuur en Waterstaa [Ministry of Health, Welfare and Sport, Ministry of Agriculture, Fisheries, Food Security and Nature and Ministry of Infrastructure and Water Management].Nederlands Actieplan voor het terugdringen van antimicrobiële resistentie 2024-2030 [The Netherlands' Action Plan for Reducing Antimicrobial Resistance 2024-2030]. Den Haage:Rijksoverheid;2024. Dutch. Available at: https://open.overheid.nl/documenten/686e0ef0-5074-4fac-aa57-f5a69d59dc7a/file
31. Government of Norway. Norwegian National One Health Strategy Against Antimicrobial Resistance 2024–2033. Oslo: Government of Norway;2024. Available at : https://www.regjeringen.no/contentassets/7ae8eacec9cc4af085b5c113a98a0eb0/national-one-health-strategy-against-antimicrobial-resistance.pdf
32. Norwegian Ministry of Health and Care Services. National Strategy against Antibiotic Resistance 2015–2020. Oslo: Norwegian Ministry of Health and Care Services;2015. Available at: https://www.regjeringen.no/contentassets/5eaf66ac392143b3b2054aed90b85210/antibiotic-resistance-engelsk-lavopploslig-versjon-for-nett-10-09-15.pdf
33. Norwegian Institute of Public Health, Norwegian Veterinary Institute ,University Hospital of North Norway. NORM, NORM-VET: Usage of Antimicrobial Agents and Occurrence of Antimicrobial Resistance in Norway 2024. Tromsø,Ås,Oslo:2025. Available at: https://www.fhi.no/contentassets/00f0b4a7a3d242c89b931c3badefdaf3/norm-norm-vet-2024-komplett.pdf
34. Gabinete da Secretária de Estado da Promoção da Saúde [Office of the Secretary of State for Health Promotion]. Atualiza o Programa de Prevenção e Controlo de Infeções e de Resistência aos Antimicrobianos (PPCIRA) [Update the Infection Prevention and Control Program and Antimicrobial Resistance (PPCIRA).]. Diário da República n.º 174/2022, Série II de 2022-09-08. Despacho No. 10901/2022 of September 8 2022, 93 – 99. Portuguese. Available at: https://diariodarepublica.pt/dr/detalhe/despacho/10901-2022-200789503
35. Gabinete da Secretária de Estado da Promoção da Saúde [Office of the Secretary of State for Health Promotion]. Altera os n.os 11 e 24 do Despacho n.º 10901/2022, de 2 de setembro,

publicado no Diário da República, 2.ª série, n.º 174, de 8 de setembro de 2022, que atualiza o Programa de Prevenção e Controlo de Infeções e de Resistência aos Antimicrobianos [Amends numbers 11 and 24 of Dispatch no. 10901/2022, of September 2, published in the Diário da República, 2nd series, no. 174, of September 8, 2022, which updates the Program for the Prevention and Control of Infections and Resistance to Antimicrobials.]. Despacho No. 6386/2023 of June 9 2023. Diário da República n.º 111/2023, Série II de 2023-06-09, 145 – 146 Available from: https://diariodarepublica.pt/dr/detalhe/despacho/6386-2023-214175433
36. Ministerio da Saúde [Ministry of Health]. Relatório Anual 2021. Acesso a cuidados de saúde nos estabelecimentos do sns e entidades convencionadas [Annual Report 2021. Access to healthcare in national Health services establishments and partner institutions]. Lisbon:2022;Ministry of health. Portuguese. Available at: https://www.acss.min-saude.pt/2016/07/22/relatorio-anual-de-acesso-ao-sns/ .
37. National Authority for Medicines and Health Products (Infarmed). Antibióticos [Antibiotics]. https://www.infarmed.pt/web/infarmed/entidades/medicamentos-uso-humano/monitorizacao-mercado/benchmarking/benchmarking-hospitalar/antibioticos Website. Last accessed 29 August 2025.
38. Public Health Authority of the Slovak Republic. Ciele pre znizenie rezistencie na antibiotika. Slovensko. Internet, accessed 13 November 2025. Available from> https://www.uvzsr.sk/documents/d/ruvztn/ciele-pre-znizenie-atb-rezistencie-ciele-pre-slovensko-do-roku-2030
39. Ministry of Health. Državna strategija »eno zdravje« za obvladovanje odpornosti mikrobov (2019 – 2024) z akcijskim načrtom za obdobje 2019-2021 [The national strategy 'one health' for managing microbial resistance (2019 - 2024) with an action plan for the period 2019]. Ljubljana: Ministry of Health;2019. Available at: https://www.gov.si/novice/nov-vlada-sprejela-drzavno-strategijo-eno-zdravje-za-obvladovanje-odpornosti-mikrobov-2019-2024-z-akcijskim-nacrtom-za-obdobje-2019-2021/
40. Government Offices of Sweden. Sveriges strategi mot antimikrobiell resistens 2026–2035 [Swedish Strategy to Combat Antibiotic Resistance 2026-2035. Stockholm: Government Offices of Sweden; 2025 Swedish. Available from: https://www.regeringen.se/globalassets/regeringen/bilder/socialdepartementet/folkhalsa-och-sjukvard/amr/sveriges-strategi-mot-antimikrobiell-resistens-20262035.pdf
41. United Nations (UN). General Assembly High-Level Meeting on antimicrobial resistance 2024. Political Declaration of the High-level Meeting on Antimicrobial Resistance. New York: UN; 2024. Available from: https://www.un.org/pga/wp-content/uploads/sites/108/2024/09/FINAL-Text-AMR-to-PGA.pdf
42. Strama - samverkan mot antibiotikaresistens [Collaboration against antibiotic resistance]. Stramas mål för antibiotikaanvändning inom öppen vård [The Strama tragets for antibiotic use in ambulatory care]. STRAMA; 2016. Swedish. Available from: https://strama.se/wp-content/uploads/2025/04/stramas-mal-for-antibiotikaanvandningen-beskrivning.pdf
43. Strama - Samverkan mot antibiotikaresistens [Collaboration against antibiotic resistance]. Nationella Slutenvårdsindikatorer för antibiotika [National inpatient indicators for antibiotics]. STRAMA; 2024. Swedish. Available from: https://strama.se/wp-content/uploads/2025/04/nationella-slutenvardsindikatorer-antibiotika-2024-05-15.pdf
44. Public Health Agency of Sweden. Antibiotikaförsäljning i öppenvård-interaktiva faktablad [Antibiotics sales in outpatient care - interactive fact sheets]. Website. Last accessed 29 August 2025. Available at: https://www.folkhalsomyndigheten.se/faktablad/antibiotikastatistik-oppenvard/
45. Public Health Agency of Sweden (FHM) and Swedish Veterinary Agency (SVA). Swedres-Svarm 2024. Sales of antibiotics and occurrence of antibiotic resistance in Sweden. Solna/Uppsala:FHM/SVA:2025. Available from: https://www.sva.se/media/amupibfr/swedres-svarm-2024-webb.pdf
